# How far is the journey before malaria is knocked out of Zimbabwe? (or Africa): a commentary

**DOI:** 10.1186/s12936-019-3053-y

**Published:** 2019-12-16

**Authors:** Clive Shiff

**Affiliations:** Department of Molecular Microbiology and Immunology, Johns Hopkins Bloomberg School of Public Health, Baltimore, MD 21205 USA

**Keywords:** Sustained malaria control, Health infrastructure, History, Local priorities and politics, Zimbabwe, Africa

## Abstract

Recent publications and statements have drawn attention to a sustainable system of managing malaria control interventions globally but especially on the Continent of Africa. Arbitrary and unstable governments often interfere with health programmes, causing upsurges in malaria transmission as well as other health issues. A well-run health infrastructure will deal with public health as a whole. This commentary follows historical conditions in Zimbabwe where much original work on malaria control was initiated and implemented and where unstable conditions happened through local politics. These periodic conditions of instability on the ground challenge the current philosophical thrust to eradication and stress the need and role of an established and well-staffed health infrastructure in each country. Such facilities should be well staffed and supplied with drugs and point-of care diagnostic tests to manage malaria and should be sustained to serve the community even after tools that can eradicate malaria are developed.

## Background

A recent paper in Malaria Journal reporting on the 2016 Malaria Indicator survey in Zimbabwe asks “How Far is the Journey to Knock out Malaria?” [1]. This rhetorical question is on the minds of public health personnel dealing with the problem of malaria in many African countries. Dube et al. present a recent malaria indicator survey which shows evidence of declines in transmission as success due to recent national anti-malaria operations in Zimbabwe [1]. The question that arises is, can this now be sustained? Malaria control in Zimbabwe as well as other African nations has fluctuated considerably over the years. The reasons are several: intermittent rainfall, drug or insecticide resistance, bad planning or perhaps as a result of local political decisions. Maybe this is a time for reflection because if one considers the past ebbs and surges of this disease on the continent, one needs to be pragmatic and address all the reasons why this happens. The World Health Organization (WHO) is setting local elimination targets, but is elimination sustainable in the light of African politics and priorities? Certainly, it is a noble goal and potentially obtainable. In Zambia this approach has drawn success in the Southern province and simultaneously expanded and strengthened the wider health care system in the country. Zambia is not unique in this approach as other countries in Africa and elsewhere have focused on local elimination and overall public health improvement.

Obviously for donors who support much of the global effort to reduce malaria it would be appropriate to see the end of this debilitating and lethal disease and they would prefer the process of eradication. With eradication the problem would be over. This target also drives much of malaria research in universities and institutes almost all of which are not lodged in endemic countries. Magic bullets of sorts would provide an end point. This has been debated extensively [2]. Recently the Lancet Commission Report has focused attention again on eradication on a global basis [3]. Whereas there has been considerable success in North America, much of Europe and elsewhere [4], it is more of a diversion of resources certainly for Africa and in that context is premature. It assumes that like smallpox, teams could swarm through the continent blotting up malaria cases. It will not happen like that; malaria control and malaria elimination requires a properly functioning health care system, a system that will be in place continuously, but staffed and operated within the purview of a Ministry of Health. These thoughts are also expressed in a news topic in Science underwritten by several of the world’s most pragmatic malariologists [5]. Surely it is best to apply good public health practice and plan on a long term health management programme especially in Africa where political priorities are unpredictable and health system sometimes lose funding. The issue of malaria eradication is not realistic at this time and should be shelved until new tools appear. We, being public health pragmatist’s, should plan on long-term management of malaria, in this way the health infrastructure will cope with unexpected outbreaks and maintain a locally run functioning health system. To this end, it helps to consider the situation in Zimbabwe which has one of the longest runs on malaria control in Africa. It has had significant success and ebbs in its history in spite of local elimination of malaria for some 30 years. It is prudent to realize that the journey to master malaria will be long and non-linear. There is an historical precedent.

## Historical experience

In the early 1950s, with encouraging preliminary results using the new residual insecticides and the effective drug, chloroquine, the WHO decided to eradicate malaria. This was successful in Europe, parts of Asia and Mauritius [4]. It was also a goal in Southern Rhodesia (Zimbabwe) because of the successful history of malaria control carried out in that country. In fact, the Federal Malaria Eradication Organization was instituted there in 1962 and planned to involve the Central African Federation block. However this did not get beyond the pilot stage and was abandoned [6]. Zimbabwe was one of the original countries that showed the potential effect of using indoor residual spraying to control malaria. An initial trial in 1949, using the residual insecticide, benzene hexachloride (BHC) was undertaken in the Mazoe river catchment in the North East of the country [7]. The work was financed not by government, but by the local State Lottery. It was shown to have a pronounced effect on transmission by recording hospital admissions for malaria among local residents in the Bindura and Shamva Hospitals [8]. The results impressed the Ministry of Health and the WHO so that a malaria control programme was expanded throughout the country. It concentrated on protecting the population in the endemic part of the country and preventing invasions of the non-endemic high veld (above 1250 m). The initial work was planned locally and commenced in 1952, and subsequently expanded to cover the entire country in 1956 [8]. Later with assistance from the WHO, professional staff were transferred from Swaziland (now eSwatini), where the operations were successful, to Rhodesia. The intervention became effective with technical and financial support from WHO and the local Federal government. Essentially the entire area below 1250 m altitude, where transmission was seasonal, was systematically subjected to regular malaria control operations using chloroquine and indoor residual spraying. The strategy was based on the seasonal application of residual insecticides and freely available anti-malarial therapy available at all clinics. All residences, sleeping accommodation and peripheral structures in compounds villages and townships were targeted to cover 70–80% of houses throughout the country each cycle [6]. Insecticides were applied by trained personnel, under careful supervision, seasonally prior to the advent of the rains usually in November.

From the 1950s, the entire malaria control programme in Zimbabwe was operated by the Ministry of Health through the Blair Research Laboratory in Harare. All scheduling, planning and procurement was carried out by personnel at the Institute. All training and operational work as well as supervision was centralized at Blair as was assessment of the operations. Supervision was done in two tiers, first by the team leader who was directed to observe the actual spraying operation and additionally and unannounced, by field entomologists. These personnel also carried out mosquito collection and took samples from the walls to assess insecticidal efficacy. Research, evaluation and entomological studies were under the same roof and direction. Altogether there were over 30 dedicated scientific personnel including entomologists, a malariologist and technical and supervisory permanent staff directly involved in the work. Sadly, the science of epidemiology had not been developed so effective assessment was missing from the local expertise and much data were lost. Field operations and supervision were coordinated with the Provincial and District medical officers and their staff.

There were operational difficulties: clinical diagnostics were difficult especially as there was no point of care diagnosis, and blood films although taken regularly at rural clinics were of little value to the staff, as patients had to be diagnosed clinically and treated immediately with chloroquine. It is unfortunate that records were not maintained and the thousands of blood films sent regularly to microscopists at Blair Laboratories were examined but not properly recorded. The burden of malaria as a disease of concern was alleviated for many years throughout the country. There was also no dream of eradication. Essentially the disease was under control and the control was dependent on a strong infrastructure operated and financed by the Ministry of Health for nearly 30 years [7]. In the changeover of government in 1980, the programme was decentralized and operational authority was passed to the Provinces and Districts, with research and assessment still in the hands of research and field staff at Blair. Much of this is discussed by Taylor and Mutambu [9]. Taylor was at that time Director of the Blair Research Institute and managed the surveillance system. All was well until the late 1990s when government priorities devastated operations in the Ministry of Health, and the health infrastructure collapsed and many professional staff were lost to emigration. The entire farming-based economy was destroyed [10], many technical and professional staff emigrated and malaria resurged. The dire results are reflected in the Roll Back Malaria Country needs assessment [11]. In the field only clinically diagnosed cases were reported. However, annually these were about 1.5 million, a situation that did not change over the previous 10 years. Sadly, misappropriation of funds that were provided by donors for IRS implementation became unavailable for any significant control. The report is an indictment of the national administration of Zimbabwe at that time. The WHO report quotes “the previously highly-regarded Health Management Information System in Zimbabwe must be re-established “, important features of any malaria control operations had failed [12]. My point is that the health infrastructure had collapsed and needed to be reinstated as indeed it was when the financial situation stabilized in 2010–2011. In reports hundreds of thousand cases of malaria occurred annually, but interventions were sporadic and ineffective (Shiff, personal experience). The recent publication from Zimbabwe is an indication that now the malaria situation has improved [1]. This type of situation with ebb and flow can happen in any of the endemic African countries and will allow malaria to resurge. In fact, in the National Health Strategy of Zimbabwe 2016–2020 annual malaria cases surged from 74,221 in 2009 to 518,030 in 2014 http://www.ccmzimbabwe.org.zwindex/. According to Dube et al. [1] there was a sharp decline in malaria by 2016 but their data derive from a cross sectional survey, not overall from annual reports from rural clinics and hospitals; one is still in a confusing situation. As of now, in Zimbabwe, recommendation was made to set the situation on a proper track and the report predicted that the Roll Back Malaria targets for 2010 could be achieved [1].

Currently, there has been a remarkable decline in the prevalence of malaria shown in this indicator survey, but one does worry about the level of planning and supervision of indoor residual spraying in the various districts as noticed by the author on a recent visit (Jan. 2019) to Manicaland. There are strong indications that proper supervision is still lacking and inadequate quantities of insecticides may be used by operatives. Comprehensive supervision in the field is essential to ensure effective coverage and use. The concept of management must involve the national government, but also donor interest in long-term work and fits well with aspects of the RBM Action and Investment to defeat malaria [13]. Bearing all this in mind and happening in a somewhat well-developed African country, one wonders whether the Lancet Commission [3] really understood all the issues that need to be faced.

## Conclusions

Keeping malaria under control, particularly in Africa requires multifaceted operations that involve Government as well as international sources of support. Historically programmes have suffered from periodical relapses. These relapses are not only technical and dependent on periodic breakdowns in the supply chain, they are also affected by local political issues even internecine or local warfare. In spite of reason such a situation has happened and will happen again. This fact needs to be kept in the picture both locally and through the WHO and other donors. Malaria control requires a good management system with easily procured and timely supplies, and with strong coordination at the centre. It is imperative that this system is supported and well-staffed as a priority of the Ministry of Health. Even with that, malaria will take a long time to be kicked out of Zimbabwe or anywhere in malaria endemic Africa.

## Data Availability

No data involved.
